# How co-locating public mental health interventions in community settings impacts mental health and health inequalities: a multi-site realist evaluation

**DOI:** 10.1186/s12889-023-17404-x

**Published:** 2023-12-07

**Authors:** Cleo Baskin, Fiona Duncan, Emma A. Adams, Emily J. Oliver, Gillian Samuel, Shamini Gnani

**Affiliations:** 1 Department of Primary Care and Public Health, Imperial College London, St Dunstan’s Road, London, W6 8RP UK; 2 Population Health Sciences Institute, Newcastle University, Baddiley-Clark Building, Newcastle, NE2 4AX UK; 3 The McPin Foundation, 7-14 Great Dover Street, London, SE1 4YR UK

**Keywords:** Community organisations, Co-located services, Mental health, Public Mental Health, Interventions

## Abstract

**Background:**

Public mental health interventions are non-clinical services that aim to promote wellbeing and prevent mental ill health at the population level. In England, the health, social and community system is characterised by complex and fragmented inter-sectoral relationships. To overcome this, there has been an expansion in co-locating public mental health services within clinical settings, the focus of prior research. This study evaluates how co-location in community-based settings can support adult mental health and reduce health inequalities.

**Methods:**

A qualitative multi-site case study design using a realist evaluation approach was employed. Data collection took place in three phases: theory gleaning, parallel testing and refining of theories, and theory consolidation. We collected data from service users (n = 32), service providers (n = 32), funders, commissioners, and policy makers (n = 11), and members of the public (n = 10). We conducted in-depth interviews (n = 65) and four focus group discussions (n = 20) at six case study sites across England, UK, and two online multi-stakeholder workshops (n = 20). Interview guides followed realist-informed open-ended questions, adapted for each phase. The realist analysis used an iterative, inductive, and deductive data analysis approach to identify the underlying mechanisms for how community co-location affects public mental health outcomes, who this works best for, and understand the contexts in which co-location operates.

**Results:**

Five overarching co-location theories were elicited and supported. Co-located services: (1) improved provision of holistic and person-centred support; (2) reduced stigma by creating non-judgemental environments that were not associated with clinical or mental health services; (3) delivered services in psychologically safe environments by creating a culture of empathy, friendliness and trust where people felt they were being treated with dignity and respect; (4) helped to overcome barriers to accessibility by making service access less costly and more time efficient, and (5) enhance the sustainability of services through better pooling of resources.

**Conclusion:**

Co-locating public mental health services within communities impacts multiple social determinants of poor mental health. It has a role in reducing mental health inequalities by helping those least likely to access services. Operating practices that engender inter-service trust and resource-sharing are likely to support sustainability.

**Supplementary Information:**

The online version contains supplementary material available at 10.1186/s12889-023-17404-x.

## Background

Public mental health interventions include non-clinical services or programmes that aim to promote wellbeing and prevent mental ill health at the population level. They are in recognition of and in response to the wide range of determinants across individual, family, community, and structural levels that affect mental health and well-being [1, 2]. Public mental health interventions are therefore diverse and include, for example, knowledge and skills training, welfare and financial advice services, legal advice, and social and peer support groups.

Co-location is a term used to refer to distinct services being delivered in the same physical space. In the field of public mental health, the evidence-base to date has focused on co-locating services and interventions within the health system, for example delivering welfare or legal advice within primary care practices [3–5]. Prior research on the impact of co-location has found that access to public mental health interventions is enhanced when delivered in a familiar and non-stigmatising space [6] and that users of services report benefit to their well-being due to improvements in their social circumstances [7, 8]. Healthcare professionals have also observed benefits, such as a reduction in their consultation rates [6, 9–11].

In the last decade, in England, a combination of various policy decisions has led to changes in the health and social care system. These changes include an increased fragmentation of services, a focus on crisis intervention instead of investment in prevention and longer-term support in the community [12] and services to improve public mental health having to operate in a context of increased demand and reduced budgets, particularly in deprived areas [13]. This has exacerbated health inequalities [14, 15] and led to an increase in the number of voluntary and third sector organisations taking on roles and functions previously undertaken by the public sector. In response, there has been an expansion in co-locating services across the health, social and community sector in the UK, as a potential solution to improve the coordination of care for individuals with complex bio-psychosocial needs and reduce the negative impact of fragmented services [16–18].

Therefore, co-located services may be able to reduce mental health inequalities. Mental health inequalities arise when there is an unequal distribution of the risk of poor mental health across society due to a range of disadvantages. For example, poverty, debt, poor housing, disability, long-term health conditions, or discrimination [19, 20]. Tackling these risk factors for poor mental health will involve a wide range of actions to be undertaken [20] and has been a focus of previous research. For instance, a systematic review identified 128 studies of intervention strategies for reducing mental health inequalities. Most interventions focused on socioeconomic factors, race disparities, and age-related issues and the most common intervention was providing psychological support [21]. Moreover, a recent mapping study identified 407 community-based public mental health interventions that were currently being delivered across England which aimed to address many risk factors for poor mental health, and demonstrated the wide range of services that were available to reduce mental health inequalities [22].

However, one of the challenges with addressing mental health inequalities is that there are inequities in access to appropriate services and those at risk of poor mental health often have less access to effective and relevant support [19, 23]. Service access is not simply about the ease at with which communities can use appropriate services, it can be thought of as a complex interaction between the characteristics of the individuals such as where they live, their economic situation and their social status and the specific characteristics of the service and how they organise their resources and respond to the characteristics of the population which they serve [23]. Therefore, inequities in service access can arise for many, often hidden, underlying reasons i.e. lack of cultural sensitivity from service providers, lack of financial resources to travel to services, community members being psychologically overwhelmed due to multiple and competing needs, socio-cultural difficulties such as stigma of mental health problems in certain populations or lack of trust in healthcare professionals due to previous negative experiences including discrimination [24].

Co-location models based in the health system uphold a prevailing medical paradigm in mental health and may have such limitations when seeking to reduce mental health inequalities. Additionally, such models might reinforce stigma by associating mental health support primarily with medical services [25]. Thus, increasingly, policy has been directed towards delivering services within communities so that people access support in the places where they live. Across England, public mental health interventions are delivered in a range of community spaces, such as libraries, faith institutions, and community centres allowing the resources, support networks, and skills locally available to be drawn on [26–28]. It takes services closer to those most affected by inequalities, facilitating their meaningful engagement and involvement in shaping services, helping to improve access and uptake [29].

Despite an expansion of community-based co-location in practice there is limited evidence concerning both best practice and the impacts of these delivery models. This study aimed to understand how co-locating public mental health services for adults in community-based settings might facilitate improved public mental health outcomes. We adopted a realist evaluation approach to build theory on co-location and guide implementation on the spread and scale-up of this model of service delivery across England and comparable contexts.

### Initial programme theories and aim

We undertook an evidence review (described below), which led to the development of three preliminary theories concerning the contexts, mechanisms and potential outcomes of co-location of public mental health services in community settings.

First, co-location can facilitate the sharing of information and funding between services, helping to develop a shared vision of success. This change, away from a competitive to a collaborative culture, could lead to fewer delays in treatment, the duplication of services, and widen engagement in services [29–33]. We postulated that this is especially pertinent to the community and voluntary sector where there are scarce resources.

Second, co-location can facilitate a ‘hyper-local’ focus and understanding of community need to help overcome barriers, such as language, and improve the acceptability, responsiveness, and the quality of services provided [34]. Responsiveness of services to community need is contingent on funding sources that are flexible and focused on long-term community development rather than short-term funding cycles and metrics that are often imposed top-down.

Third, there is societal stigma towards mental health and public mental health services with many users of services reporting negative experiences, often discriminatory, when accessing statutory services [34, 35] Community co-location could provide a ‘safer’ environment to access services and be delivered in a way that reduces feelings of fear or shame and be perceived as non-stigmatising and discriminatory.

Through scrutinising these theories, we sought to close the knowledge gaps on whether and how community co-located services impact on public mental health, reach those most in need and evaluate their present and potential role in helping to reduce mental health inequalities. Overall, the objectives of this study were to: (i) understand the contexts, mechanisms and potential outcomes of co-location of public mental health interventions in community settings, and (ii) understand the mechanisms for how community-based co-located services can reduce mental health inequalities.

## Method

### Rationale for using realist evaluation

Public mental health interventions are complex and diverse and recognise the range of social and environmental factors that affect individuals’ mental health [1]. Interventions vary in their delivery and in who provides and funds them. Thus, evaluating such interventions requires an understanding of the causal mechanisms within a broader socio-political and economic context and is well-suited to realist evaluation methodology, which examines ‘what works, for whom and under what circumstances’ [36, 37]. In this case, how co-location within community spaces affects public mental health outcomes and how this varies both between individuals and between sites with differing operational and funding models. This is key for extending existing literature beyond understanding who health-based models work for (and insight to why and how) to a broader perspective on understanding how diverse models of co-location might (and might not) work. RAMESES-II [38, 39] reporting standards for realist evaluations were used.

### Case study site selection

This study aimed to develop a theoretical framework for the co-location of public mental health services within community spaces. To do so, we collected data from six different case study sites across England: Northwest London, South London, Northwest England, Northeast England, Northamptonshire, and Newcastle upon Tyne (Table 1). Sites were purposively selected to represent different geographical regions, models of co-location, services, and target populations.

**Table 1:** Case study sites

#	Site location	Type of co-location	Aim of services	Target population
1	Northwest London	Community hub	To build capacity among individuals and groups to live more effectively within the wider community	Minority ethnic populations (Somalian & Arab), migrants & refugees
2	South London	Talking therapy service and a confidential advice service co-located within foodbanks	To provide emergency food to local people who experience financial hardship and to offer expert confidential support and advice to help them resolve their financial issues and/or improve their mental wellbeing.	Members of the local community who are experiencing financial hardship.
3	Northwest England	Several services including welfare advice, advice for people aged over 50years, IT access, and social activity groups co-located within a library	To provide services to support and improve the mental health and wellbeing of people in the local community.	Members of the local community
4	Northeast England	Multiple services aimed at improving the wellbeing of the local community operated under one umbrella organisation	To provide a network of support and to address isolation and poverty in the community.	Local people and organisations
5	Northampton	Physical activity, sport and art-based services co-located within a heritage site with green and blue space	To provide a range of activities and experiences to improve health and wellbeing in Northampton town and Northamptonshire.	Members of the local community
6	Newcastle upon Tyne	User-led collaboration between mental health service users and the voluntary and statutory sectors	To enable people to use their lived experience for the benefit of others and to empower themselves in doing so.	Individuals with lived experience of mental health problems

Table 2 displays the inclusion and exclusion criteria used for case study site selection; these criteria align with recent conceptual definitions of public mental health services and were refined by the research team (which included people with lived experience of poor mental health) [25, 29, 40]. Two of six case study sites were purposively sampled from public mental health interventions previously identified in a mapping study (n = 407) [22]. Four case study sites were also identified through wider stakeholder networks. To produce a breadth of cases to inform our programme theory we purposively selected sites varying in geographies, target users, modes, and operational platforms.

**Table 2:** Inclusion and exclusion criteria of case study site selection

	Inclusion		Exclusion
Community centred	The primary goal of the service is the well-being and/or social/ cultural interests of a specific **community** (either people living in the same local area or with a particular shared characteristic)		· Treatment service · Patient-only service · Entirely for-profit activities
Goal of intervention	Primary aim of at least one of the services is to protect and/or promote an individual’s mental health and wellbeing		· Clinical care (e.g., medicine or drugs or psychological therapies) · Improving mental health and wellbeing is not the primary aim of at least one service · Virtual services that are not interactive (e.g., repositories of information)
Co-location of two or more distinct services	*Distinct services defined as*: - offering different provision - independently accessible - focused on different mechanisms/outcomes Note – may be branded separately or under one umbrella branding. *Co-location defined as*: Formal or informal interaction between services, staff and/or users Including online services		· Multi-component services · No interaction between services
Population	Service primarily aimed to support adults aged 18–65 years		Services that primarily aimed to support people aged below 18 years or above 65 years

### Recruitment process and sampling strategy

We worked with at least one co-located community organisation at each case study site. The researchers had a key point of contact at each to help inform the recruitment strategy (Table 3). To capture different experiences of co-location between providers within the same organisation, we interviewed both individuals with management roles and those involved in the face-to-face delivery of services. This included paid staff members as well as volunteers. Where possible, participants were purposively selected to ensure a diverse group based on ethnicity, gender identity, disability, age, and use of co-located services.

**Table 3:** Recruitment strategy by case study site (N = 6)

#	Service providers	User of Services
**1**	Site identified by a contact of the research team. All full-time providers were interviewed.	Recruited via service providers who translated all documentation.
**2**	Key point of contact identified on website. Further providers were contacted via snowball sampling.	In-person recruitment in two foodbank sites with help of staff and volunteers.
**3**	Site identified and contacted via existing contacts of the research team. Key point of contact recruited participants.	Recruited via providers.
**4**	Site identified via information collected in a previous study. Providers recruited via website (n = 2), snowball sampling (n = 3) and in-person (n = 1).	In-person recruitment (n = 5) and recruitment via providers for welfare advice service users (n = 3).
**5**	Site identified and all providers recruited via participant of first expert panel workshop (who was also a service provider). Member of co-creation group recruited via attending project steering group meeting.	Service providers sent study invite to all service users. No service users were recruited from this site.
**6**	Site identified by a contact of the research team. Key point of contact provided emails of staff.	Service providers sent study invite to all service users.

### Evaluation design

**Fig. 1:**
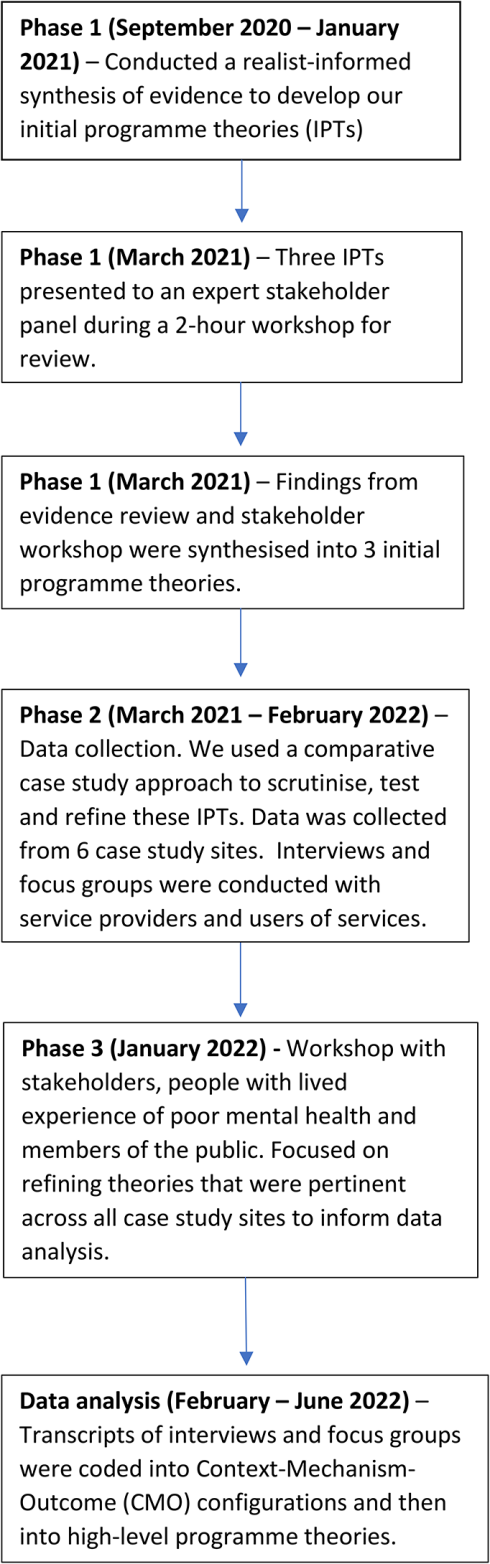
Timeline of phases and activities of research process

We adopted a comparative case study design using qualitative methods. A case study approach offers the ability to examine different models of co-location across the country and in diverse contexts [41]. Qualitative data were deemed most appropriate to explore underlying explanatory mechanisms of how co-location impacts on public mental health outcomes. Quantitative methods were not used in this study because: (1) there is inconsistent and limited high-quality data collected across community organisations [22, 27] and (2) such data offer limited insight concerning *how* public mental health outcomes are achieved, which was the primary purpose of this research study. A timeline of the research process is presented in Fig. 1.

### Data collection

Data were collected between March 2021 and February 2022. Manzano’s [42] three-part approach to realist evaluation data collection was used to guide the three phases of data collection and analysis: theory gleaning, parallel testing and refinement of theories, and theory consolidation.

#### Phase 1: identifying the initial programme theory (theory gleaning)

In realist methodology, inductive and deductive logic are used to develop initial programme theories, which are in turn used to guide subsequent data collection. A realist-informed evidence review was conducted to create initial program theories (Sect. 1.2)., This was not intended to search an exhaustive list of evidence, or publish the evidence review as its own work but to stop extracting data once consistent themes had emerged that could be used to guide subsequent data collection [43]. CB & FD examined published reviews, policy documents, implementation guidelines, and national UK websites in the field of mental health, and on co-location of services in healthcare and community settings using online databases, search engines, snowball referencing, and papers recommended by colleagues who work in the field. Plausible programme theories were coded into an extraction framework developed in Microsoft Excel, consisting of author/year; title; publication; context; mechanisms; and outcomes.

The evidence review resulted in a breadth of programme theories, which were presented to an expert stakeholder panel (n = 5; roles included wellbeing project coordinator, national leadership role in the voluntary sector, public health specialist at a local authority, senior practice lead specialising in mental health, and grant funding manager). A two-hour workshop was held to prioritise, critique, and refine these theories and additional data were collected on the policy, practice, theory, and benefits of investing in co-location models of public mental health interventions. The workshop discussion was recorded and transcribed professionally and analysed and coded for plausible programme theories into the same extraction framework as the evidence review.

Findings from the evidence review and stakeholder workshop were collectively compared, seeking both confirmatory and contradictory findings, and synthesised into our initial programme theories (presented in Sect. 1.2). Further refinement of the programme theory was iterative throughout data collection; the research team held monthly meetings to discuss emerging theories.

#### Phase 2: parallel testing and refining of theories

*Testing programme theories.* We collected information from service providers and users of services. Providers participated through semi-structured interviews. To widen participant engagement and enhance data among users of services, alternative data collection methods were offered to encourage reflection, facilitate communication, and express tacit knowledge [1, 3]. These methods included drawing or written responses to questions, however, no participants opted to use these methods. In interviews, initial programme theories were not presented directly to participants but acted as a guide for open-ended questions that related to how co-location may have impacted on service access, its delivery, and personal outcomes that related to mental health and wellbeing. Interview guides were adapted for each individual case study site. We iteratively adapted questions to account for the evolution in our initial theories and to include emergent theories.

Interviews were a maximum of 60 min to manage demand on services during a challenging period for delivery (Covid-19 pandemic-related). Sessions were conducted virtually (either by telephone or using the videoconferencing software Zoom or Teams) and face-to-face to accommodate individuals’ time constraints, availability of internet and computer or smart phone, and Covid-19 pandemic protocol measures.

*Refining programme theories.* We conducted up to two focus groups (between one to two hours in length) per study site (one for service providers and one for users of services) to further refine the programme theory. Participants who had been involved in the interviews were invited to attend the focus group discussion to consider and expand upon site-specific theories.

#### Phase 3: consolidating the programme theory

A second workshop took place after completion of case study site data collection and analysis. This was a multi-stakeholder workshop; participants included experts, people with lived experience of poor mental health, and members of the public. Individuals with lived experience of using services were invited by placing an advertisement in the McPin Foundation Involvement Bulletin [44]. We recorded demographic information from applicants to gain a sense of whether we have engaged a variety of individuals. The multi-stakeholder workshop focussed on refining the theories that were pertinent across all case study sites. Views were sought on emergent recommendations and how best to disseminate findings to policy makers, practitioners, and the public.

### Data analysis

In line with the realist approach, data collection and analysis took place in parallel [37]. Audio recordings of interviews, focus groups, and expert panel workshops were professionally transcribed. Transcripts were coded into context-mechanism-outcome (CMO) configurations [39] by CB and FD and then into high-level programme theories, which were used as the main structure for our realist analysis.

CMO configurations are the standard analytical framework on which causal explanations are built in realist evaluations [39]. They refer to a theory on how specific contextual factors (C) trigger underlying causal mechanisms (M), and how this combination generates various outcomes (O) for different people. Mechanisms describe the interaction of a resource that is offered to a person (a programme will offer an opportunity or a constraint of some kind) and how that person chooses to respond to this resource (resource + response) [37]. CMO configurations are particularly useful for evaluating co-located services as they can help us to understand the complexity of such services and how individuals’ responses to services, and therefore the effectiveness of the service, may vary depending upon specific circumstances.

We followed Maxwell’s [4] categorising and connecting strategies for data analysis by first analysing separately each case study site and then comparing evidence across sites. This was to determine how the same causal mechanism may in different contexts produce the same or different outcomes. Data were analysed on Microsoft Word; broader themes were firstly identified within each case study site and then the specific context, mechanisms, and outcomes were coded for each theme. The CMO configurations within and across sites were then compared. Initial programme theory hypotheses were revisited and refined and/or rejected where applicable.

### Ethical approval

This study received ethical approval from the Imperial College London Research Ethics Committee (21IC6576) in accordance with the ethical recommendations of the Helsinki Declaration. Durham University Department of Sport and Exercise Sciences Research Ethics Committee granted ethical approval for the two expert panel workshops (SPORT-2021-01-05T11:06:06-lxkc61).

## Results

### Details of participants

*Theory gleaning expert panel workshop.* The online workshop included five participants (n = 4 female; n = 1 male; n = 5, all White British) from a range of professional backgrounds and seniority levels (e.g., coordinator, specialist, practice leads and managers). *One-to-one semi-structured interviews.* A total of sixty-five interviews were conducted with users of services (n = 32), service providers (n = 32) and a member of a study site co-creation group (n = 1).

*Focus group discussions.* Four focus groups were conducted (n = 20) at three sites (case study sites 1, 3 and 4). Two in-person focus groups were held separately with service providers and service users at site 1. An online focus group held at site 3 was among service providers. One in-person focus group was held at site 4 with service providers. All focus groups were comprised of participants who took part in the interviews.

*Theory refining multi-stakeholder workshop*. Fifteen participants (female n = 12, male n = 5) participated in the theory refining workshop; nine members of the public and six members who were policy makers or were involved in the design, commissioning, and/or funding of co-located services. Members of the public were White British (n = 5), Asian British-Indian (n = 2), Black or Black British (n = 1) and one preferred not to say. Two were unemployed, two had long-term health conditions, two worked part-time and one was retired.

### Overarching programme theories

This study aimed to (i) understand the contexts, mechanisms and potential outcomes of delivering public mental health interventions in community settings and (ii) understand the mechanisms for how community-based co-located services can reduce mental health inequalities. Analysis of one-to-one interviews, focus groups, and workshops elicited five high level programme theories each of which addressed both of these research aims. These programme theories encompassed 10 CMO configurations common to all case study sites (Table 4). These high-level programme theories were: (1) provision of holistic and person-centred support; (2) reducing stigma toward mental health and public mental health services; (3) delivery of services in psychologically safe environments; (4) overcoming barriers to accessibility; and (5) enhancing the sustainability of services. Most of these CMO configurations and programme theories explained how co-location of services could lead to positive outcomes, however we also included some “dark clauses”, which acknowledge the ways in which co-location may not work effectively and/or lead to negative outcomes (Table 4).

**Table 4:** Summary of Programme theories

Programme theory 1: Provision of holistic and person-centred support		
Context	Mechanisms	Outcomes
- Fragmented service provision - Community members face complex psychosocial challenges	- Regular and informal interactions between providers - Specialised support tailored to the needs of the community.	**Service providers**: - Coordinated service provision - More effective signposting - Improved effectiveness to solve complex problems **User of Services**: - Increased trust - Improved awareness & engagement - Increased ability to maintain own health and wellbeing and stay well long-term.
- Statutory services take deficit-based approach to support.	- Services available without a predefined level of need. - Ethos and culture empower users to be independent. - Expertise in navigating community support and local health and social care systems.	**User of Services**: - Increased ability to maintain own health and wellbeing and stay well long-term. - Increased prevention and promotion of mental health and wellbeing. - Increased trust in services and service providers.
- less bureaucracy and hierarchy	- Time to spend with service users to proactively notice issues and tailor support - Culture of being experimental and innovative with service delivery, which is often led/influenced by the community. - The unstructured nature of service provision could lead to logistical challenges**	**User of Services**: Increased ability to maintain own health and wellbeing and stay well long-term. **Service providers**: - services are more flexible and responsive to changing needs in the community - Services filled gaps and failings in existing provision - services are culturally specific/ appropriate
- Different goals and values between services	- ***Lack of communication between services***	**Service providers**: Sub-optimal signposting and information sharing
**Programme theory 2: Reducing stigma toward mental health and public mental health services**		
**Context**	**Mechanisms**	**Outcomes**
- Fear/ Negative and/or inaccurate perceptions of statutory services - Stigma associated with mental health and help seeking - Limited mental health literacy	- Distinct from statutory services. - Services delivered in a familiar setting - Providers from the community and were not gatekeepers - Distinct from mental health and formal services. - Visibility of peers needing and receiving support reduced stigma. - ***Contingent on having reputation of confidentiality***.**	- Increased and earlier access to support
- Statutory services are rigid in their expectations of service users.	- Services had little obligations, commitments, or expectations which created a non-pressuring environment. It increased feeling of ownership of a space, autonomy, agency, and choice.	- Increased autonomy - Increased service use engagement - Increased and earlier access to support-
**Programme theory 3: Delivery of services in psychologically safe environments**		
**Context**	**Mechanism**	**Outcome**
Statutory sector services felt cold and clinical with staff experiencing burn-out and empathy fatigue.	- Culture of friendliness and safety; user of services felt treated with respect and dignity - Physical environment is informal, colourful, and personal creating a sense of comfort, warmth, and familiarity.	**User of Services**: - Increased ability to maintain own health and wellbeing and stay well long-term. - Increased engagement with services - Overcoming stigma toward mental health and public mental health services - Increased trust in services and service providers
Individuals with different mental health and wellbeing needs in a shared space	- ***Environments did not always feel safe for or with service users facing acute crises***.**	- Service is less likely to be used
**Programme theory 4: Overcoming barriers to accessibility**		
**Context**	**Mechanism**	**Outcome**
- Users faced complex challenges yet service provision was fragmented - Psychological barriers limited access and engagement with services	- Cost and time efficient. This may be especially important for service-users who have disabilities or pressures on those resources. - Proximity, visibility, and cross-service signposting reduced psychological barriers to accessing services.	**User of Services**: - Increased service access and engagement
**Programme theory 5: Enhancing the sustainability of services**		
**Context**	**Mechanisms**	**Outcomes**
Short term, competitive and limited funding	- Pooling and redeployment of resources. Pooled capital costs and administration resources E.G., funding applications. - Collective reputation and impact increased chances of funding. - Services integrated within community assets.	**Service providers**: - Efficiency - Resilience - Responsiveness to need

** ‘ ***dark’ clauses/rival theories***

### Programme theory 1: provision of holistic and person-centred support

We developed three CMO configurations that underpinned how public mental health services co-located in community spaces were able to support individuals holistically and avoided adopting a narrow focus on their condition by considering their preferences, wellbeing, cultural background, and social circumstances.

#### CMO configuration 1.1: integrated and comprehensive support for complex challenges

Austerity measures to reduce public expenditure in England, welfare reform, and specialisation of healthcare professionals have contributed towards the provision of fragmented and siloed services (context). This is particularly problematic for individuals with complex needs and frequent psychosocial challenges (context). Co-location facilitates regular and informal interactions between services (resource) enabling them to learn about each other (response) to design coordinated services and achieve more effective signposting(outcome):

*“It’s a holistic approach, we are not dealing with just benefits or just debts or just housing.*

*We are identifying those multiple, linked arrears and*.

*enquiries, and then we can link them together and prioritise”. [Service provider]*

*“It’s not only brilliant for the clients and the guests that come in, it’s brilliant for us as well as*.

*volunteers and staff because you get to know a lot more about these services by having them*.

*there….you’re a lot more helpful to the guests that are coming in*.

*because you do understand which issues other people can help with rather than just*.

*signposting around”*.

*[Service Provider]*

Users of services reported that this helped improve their awareness and engagement with services and trust in service providers (outcome). Staff and users of services felt that it was more likely that problems would be resolved or progressed if services were co-located (outcome).

“[co-location] *stops all the leg work. I’m 58 now, and I can’t manage the walking around to different places, so when you come to one place and you can get it all done at one time, in the morning, or in the afternoon, it makes it a lot easier…*” *[User of Service].*

A potential challenge of co-location was highlighted at one case study site where the relationships between the staff of different services had become strained due to different goals (context) and ways of working and a lack of communication between the services (resource). This resulted in sub-optimal signposting and information sharing (outcome).

*“I do think it comes down to a willingness of all parties to work together…because then it forms an understanding of issues about the building, its set-up, the way that people work, and the procedures that everybody has in place. I will say that for us and the relationship with [co-located service], they very much operate on their own, they’ve got their own set of clients. They deal with them in a certain way, which hasn’t always been helpful to us. I would prefer to do much more, where we can operate in more of a partnership way” [Service Provider]*.

#### CMO configuration 1.2: proactive and preventative ethos

Current statutory service models for mental health services typically offered support when someone was acutely unwell (“*It’s almost saying, go away until you feel worse and then come back” [Service Provider];* context). Community organisations had open services with no predefined level of need (resource) accessible before an individual reached a crisis point (response). The ethos and culture of the services were focussed on empowering users to access help and be independent (resource) by equipping them with the confidence, knowledge, and skills to manage their own health and wellbeing more effectively. Services offered expertise in navigating community support and local health and social care systems (resource) and connected people to community resources, information, and social activities (response).

*“There is still a tendency in the NHS to see mental health in terms of time-limited interventions. We work from the starting point that nobody has just mental health problems. Loads of people who are coming to us exclusively for their mental health, but there’s always other stuff in there. There’s a load of drug and alcohol issues, obviously. There’s quite a lot of autism spectrum disorder, or a learning disability, or ADHD. A lot of physical health problems, long-term conditions. Medically unexplained symptoms. And a ton of poverty and housing issues as well. So, we need to be equipped to deal with all of those simultaneously and not say, well, you come here for this and then the rest of it’s not our concern” [Service Provider].*

Consequently, users of services gained trust in providers and perceived them better at promoting mental health and wellbeing and preventing mental illness (outcome). As a result, they had increased confidence and autonomy to manage life challenges and stay well long term (outcome).

*“I’m a mental health patient,, so when I’m not coping, I can come here and get a nice smile from somebody, and a helping hand, and sort myself out and go away. They’re doing l what the hospital should be doing for me, they help me out, and they sort me out, and I go back on my way and I’m able to cope for a little bit longer” [User of Service]*.

#### CMO configuration 1.3: flexible and tailored service provision

Community organisations were smaller, less bureaucratic, and hierarchical than statutory services and therefore had greater societal permission and ability to be flexible and experiment with how they deliver services (context). This meant that they could spend time (resource) to build trust, proactively identify problems, and tailor their support to individuals (response) who felt listened to and supported (response). Providers were enthusiastic and empathetic individuals with a strong commitment and understanding of the local area and community (resource), which contributed towards creating a culture of services that were responsive and appropriate (response). The structure and culture of community organisations increased their ability to be flexible, experiment, and innovate in how they delivered services, which was often led or influenced by the community (response).

*“Being a small organisation, there is a lot of freedom because you don’t have a big bureaucracy bearing down on you. …. I think in the community sector, people have got more experience of being able to adapt very quickly…, we’re not sitting around waiting for the latest guidance to come out, we just go out often and do something.” [Service provider]*.

As a result, services were more responsive to changing needs in the community, more culturally specific and appropriate, and filled gaps, and failings in existing service provision (outcomes).

*“I think it’s better to come here because they’re more geared to what you’re going to be asking for, and the questions that you’ll be asking, and the things that you need” [User of service].*

However, we found that the unstructured nature of service provision could lead to logistical challenges such as not being able to guarantee numbers that will use a service on any given day.

“*there were some sessions where two team members would come and would sit and actually there was no one who was wanting to talk to them that day. … but then the next time, it could be a really fruitful time where we’ve talked to ten-plus different people”* [*S*ervice provider].

### Programme theory 2: reducing stigma toward mental health and public mental health services

We found significant levels of mistrust and fear towards statutory providers and formal mental health services. We found two CMO configurations that underpinned how co-locating public mental health services in community settings has helped dispel fear associated with mental health and address stigma in seeking support.

#### CMO configuration 2.1: distinct from statutory services

Many people felt let-down by formal statutory and mental health services and were unwilling to access services due to discrimination, fear of negative judgement, and stigma that meant people sought support only when in a crisis (context). Co-located services within a community space (resource) felt safer, familiar, and comfortable with services continuing to adapt their provision to local need (response). They provided a ‘softer’ access point for individuals to receive support among peers in shared circumstances (resource) and detached from associations with mental health or clinical services, which reduced feelings of shame and encouraged people to seek help (response).

*“voluntary services, because they don’t have those statutory powers, they can present as less threatening and coercive” [Service Provider]*.

By having diverse access points to receive support (resource), users felt greater ownership and agency (response) and after brokering trust, providers were able to explore sensitive topics and refer users if needed (resource) to more intensive or appropriate services (response). These mechanisms widened earlier access to support, especially among those not in touch with formal services (outcomes).

*“I think it is just a lovely, safe place for everybody. You know that you can walk through the door and there’s no judgement. If you want to sit and not have a conversation, it’s absolutely fine. When you do your classes and drop-ins there’s no pressure”. [User of Service]*

#### CMO configuration 2.2: informal access and engagement opportunities

Statutory services can have strict eligibility criteria with often high service thresholds for individuals to access services whilst users of services need, and prefer, more flexible services that are in informal settings (context). Without a gatekeeper role (resource), community providers were able to establish relationships with more equal power dynamics and contact that felt less transactional to users of service (response):

*“Just being able to come somewhere and have some social interaction without needing to have made a booking or explain who you are and why you’re here” [Service provider]*.

Community organisations had fewer obligations around how services were used (resource) which created a less pressured environment (response) and where people who used the services felt greater autonomy and agency in how they received support (response).

*“If you want to quietly slip away from an activity, you can do. You’re not stuck in the room feeling pressurised that you’re in this uncomfortable environment. It’s very open. It’s a lovely community, cosy atmosphere.” [Service provider]*.

In addition, there were clear benefits to some elements of support not being labelled explicitly as mental health related. Collectively, this increased engagement with services, and helped those who used the service to be better able to self-care, especially for some individuals who were managing challenging mental health related symptoms. (outcome).

*There’s no, this is a mental health activity tag attached to stuff. But things naturally have that ability to improve people’s mental health and to improve their lifestyle perhaps. But there’s that much on offer that you can cherry pick what’s right for you and what you need to help improve the way you’re feeling” [Service provider].*

### Programme theory 3: delivering services in psychologically safe environments

Users of services were often fearful and uncertain about their health and social circumstances when seeking help from services. The environment, both physical and relational, contributed towards a stressful situation. We found that community-based co-location of services facilitated a positive, friendly, and relaxed environment through two CMO configurations.

#### CMO configuration 3.1: friendly environment

Due to resource pressures and a performance-focussed culture within the statutory sector, staff themselves were stressed with many leaving their job (context). On the other hand, community providers including paid workers and volunteers (some of whom had previously used the services) were able to use their shared life experiences to create a culture of friendliness, empathy, trust, and collaboration (resource). They had the time and ability to ensure that the first point of contact was warm and welcoming (resource). People who used the services felt they were treated with respect and dignity, and it was a place of support and empathy (response). They felt less socially isolated and that the burden of their problems had been lifted (response). The trust brokered in this environment was transferred to co-located services within the same space, which increased overall levels of engagement (outcome).

*“And just really having that core of trying to treat people with dignity and respect and try and create this place where they’re valuable and they’re very safe. And it’s non-judgmental”*. *[Service provider]*

“*Not only do they sit down with you, but they want to be here.*

*In the jobcentre, they don’t want to be here. They’re just there for their nine until five.*

*It’s just a job they have found… Here, they care. For what reason*,

*I don’t know. I’m just a stranger to them, but they clearly care for some reason. Whatever reason, I enjoy it.” [User of Service]*.

However, there are disadvantages to co-locating services in communities with diverse needs. Such environments may not be well-suited to supporting users of services experiencing stressful or distressing circumstances (resource); in turn observing others’ distress, anger, or frustration was noted as something that undermined the experiences of other users of the services (e.g., they felt annoyed, irritated, stressed, scared, embarrassed, and/or threatened (response)). Consequently, this meant that some people were less likely to use the service (outcome).

*“I suppose it depends on what services you’re putting together. And sometimes you want those services to gel because the users of them would really benefit from using both services. But it is sometimes at the detriment of other service users….we’ve had lots of customers. And they’ve said I’m not going in there again, because the behaviour that I’ve been exposed to from customers from some different organisations that are based in there is just outrageous and it’s threatening and it’s intimidating, and I’m not using that service again*”. [Service provider].

#### CMO configuration 3.2. warm and welcoming physical environment

User of services reported that the delivery environment of statutory services was cold and clinical. Meanwhile the décor in community spaces was informal, colourful, and personal (resource) which created a sense of comfort, warmth, and familiarity (response). This led to better engagement with services and an improved sense of independence and empowerment.

*“You want it to have a warm feel to it, colourful things on the walls and things to look at. Something to be a bit uplifting about the place, not just a cold miserable building because I think if I walked into somewhere like that I’d probably walk back out again.” [Service Provider]*.

*“Even though people are struggling, it’s just so calm. It’s brightly coloured…you would walk in and there’s fairy lights up and things…. they’re really trying to make it look like a happy place.” [User of Service]*.

### Programme theory 4: overcoming barriers to accessibility

Fragmented services can be a challenge to navigate and physically access, time consuming, and costly, especially for people with disabilities or complex, multiple needs, or those with limited resources, or cost, and time pressures (context). This can reduce an individual’s psychological and emotional capacity to seek support (context). Accessing co-located services was less costly and more time efficient. The visibility of services (resource) reduced psychological barriers related to the anxiety of something new and stress about suitability of services (response). This meant that people accessed services that they would not normally (outcome) and it increased service access and engagement with people in crisis or with multiple complex problems (outcome).

“*The partnership helped them to access a whole sphere of society that they knew they were missing beforehand… I think going somewhere new is always often quite a daunting thing,, hard to find the right place and which bus to get and how to get there and what it’ll be like. particularly if you’re experiencing mental health difficulties.*” [Service provider].

“*Because when you have to travel between places it can be frustrating, can be difficult.*

*Financially, it’s not feasible because going back and for and then waiting, you just don’t have time. And then you just feel like, if you get there and it’s not as beneficial as you want it, you feel deflated. You don’t want to bother using the service again… Whereas here, I had to come here for more than one reason.*” [User of Service].

### Programme theory 5: enhancing the sustainability of services

Our fifth programme theory, consisting of two CMO configurations, was related to the sustainability of services.

#### CMO configuration 5.1: pooling resources

Community organisations typically operate under limited resources competing for short-term funding (context). Co-located services with integrated resources- administrative, capital, insurance costs, office and venue space and equipment, and human resources-as well as volunteers and other community assets (resource) were better able to share the financial risk (response) and spend more time focussing on delivering services or seeking additional funds (response), which in turn helped improve overall efficiency in resource use (outcome).

Although co-located services were better placed to ensure business continuity when circumstances forced change in services delivery (response) and improved their response to shocks in the system, for example climate related events or public health emergencies (outcome), the dependency on a volunteer workforce was also considered a vulnerability.

*“My job would be a nightmare because I wouldn’t be able to do the things I do if I had to spend at least 50% of my time trying to find funding to keep us going. Independently, we could probably exist, but on a much, much, much reduced scale and be a lot less effective.” [Service provider]*.

*“I think having the umbrella organisation, instead of having 12 different charities all competing for the same money, all competing for the same volunteers, all competing for the same board of trustee members, we’re all under that one umbrella.” [Service provider]*.

#### CMO configuration 5.2: increased reputation and impact

Both the reputation and impact of co-located community services compared to individual services was greater from the perspective of funders and community members (resource). This increased the chance of receiving funding (response) enhancing resilience against shifts in the funding landscape (outcome). However, the sustainability of services was contingent on having good long-term relationships with the council and funding bodies, as well as with each other. Service providers highlighted that effective integration of services requires more than just physical co-location:

*“. There needs to be a commitment, and more than just a paper commitment, to working in a different fashion. There needs to be a cultural fit, and also, an operational fit in the way that you’re delivering these services. Otherwise, then you’ve just got, yes, the silos are closer together physically, but you haven’t broken down the barriers.” [Service Provider]*.

## Discussion

### Summary of findings

We used realist methodology to understand (i) the contexts, mechanisms and potential outcomes of co-location of public mental health interventions in community settings and (ii) whether and how community-based co-located services can reduce mental health inequalities.

#### Co-location of public mental health interventions in community settings

Our initial programme theories that community co-location would facilitate resource-sharing, responsiveness to local needs, and a less stigmatising way to access mental health services were supported and extended. Specifically, our findings provide detailed evidence of how co-locating services and interventions in the community is better able to provide holistic and person-centred support for individuals facing multiple and complex issues than statutory or more formal services. This concurs with the widespread consensus and legislative push to integrate services in the health, social and voluntary sector [45, 46]. We found delivering public mental health services in community settings facilitated a culture of service delivery that was less bureaucratic and hierarchical than in some statutory organisations. Therefore, there was greater flexibility to innovate and experiment with how services are delivered so that they are tailored and responsive to the needs of the local community. Users of co-located services in the community had more variety in where they could access support and did not have to meet or follow specific access criteria, ultimately increasing autonomy and agency in how, when, and if they use a service.

However, we also found that there were some challenges, specifically surrounding conflicts of interest between users of different services within the same space and service providers having different goals and values. This issue had led to people being discouraged from using a service as well as straining the relationships between the staff and volunteers of each service.

#### How community-based co-located services can reduce mental health inequalities

Our findings suggest that the main mechanism by which community-based co-located services can reduce mental health inequalities is by improving *access* to services which address the risk factors for poor mental health (debt advice, housing support, employment and other socio-economic factors) by providing solutions to some of the hidden obstacles to service access. For instance, we found how co-located services were able to overcome psychological barriers (such as anxiety, stress and feeling intimidated) to service access. The proximity of services reduced the amount of time and effort required to access multiple services and therefore significantly reduced stress amongst service users with multiple needs. The warmth, friendliness, and empathy that the volunteers and staff offered made asking questions about other services less intimidating and therefore reduced uncertainty about the suitability of a service. It enabled staff to support service users in their transition between and across services and therefore reduce the stress of these transitions.

A further key finding concerned the role of trust and empathy as an underlying mechanism for increasing access to services and therefore reducing mental health inequalities. In the healthcare literature, trust has been reported to be associated with patients being more likely to be open and honest about their health [47], improved patient outcomes [48], increased likelihood of using services on a regular basis and greater adherence and satisfaction from patients [49]. A healthcare provider’s communication style, interpersonal skills, ability to be warm and receptive and treat their patients with respect and dignity have been reported as characteristics which can increase patient’s trust [50].

However, many studies have reported a decline in such empathetic skills amongst healthcare professionals [51–54]. Likely factors in the reduction of empathy include long working hours, staffing shortages, inability to spend sufficient time with patients, increased pressure to meet operational targets and the high number of patients that professionals have to manage [50, 55], which are common characteristics of many statutory and health services. Community co-located organisations, through adopting an organisational culture that promotes and values empathy and friendliness, can engender a more trusting relationship with their communities. This enables them to reach people who may not be accessing statutory or health services due to fear of negative consequences or previous negative experiences. Although, it should be acknowledged that the staff and volunteers of community organisations are not immune from experiencing compassion fatigue [48] or feeling the need to emotionally distance themselves from people in complex and distressing situations to protect their own mental health [57].

Relatedly, we found significant levels of mistrust and fear towards statutory providers and formal mental health services. Our findings indicate that providing services within local communities and by individuals with shared life experiences reduced the risk of negative experiences, and fear and stigma among those accessing the services. Thus, reducing mental health inequalities by making service access easier. However, it was important for services to have a reputation of being confidential and free of judgement. This is consistent with previous research which reported that the relationship between service providers and service users influences access to mental health services [58–60]. All these relationship mechanisms are likely to support earlier and more frequent engagement in support for those facing risk factors for poor mental health who often present late to health services, contributing to the prevention agenda and reducing mental health inequalities.

### Strengths, limitations, and future research

This study has several strengths. We examined sites from different regions in England, some delivered by non-traditional (i.e., non-statutory) providers, which offered a wide variety of services and used different models of co-location in different settings. These settings are often under-evaluated [27], so the research contributes to an emerging evidence base concerning community-delivered service models. However, due to the diverse range of types of services that could potentially be offered in a community organisation and diverse ways in which a service can be delivered, our results do not, and are not intended to capture nor represent all instances of co-located services within the community. We present our developed programme theory as an overarching framework for refinement and expansion; we note that none of our sites were delivered in predominantly rural areas and highlight this as a focus required in future work.

It should also be noted that we sampled those using services as opposed to those who might need services, potentially biasing the perspectives of those who have favourable opinions and/or experiences of these services. It should also be considered that service providers may have been reluctant to discuss negative aspects of their services due to fear of jeopardising future funding, wanting to maintain social desirability to the interviewer, or not wanting to be disloyal or disrespectful towards the service where they work or volunteer. To mitigate these concerns confidentiality was assured to both service users and service providers and the interviewers specifically asked every participant if they perceived there to be any negatives of the co-located service that they provided or used. Future work mapping reach of community co-location versus population need in communities is required.

Due to the cross-sectional nature of the study, we are limited in our ability to reflect on the longitudinal impact of these services, despite some service users speaking about changes over time. The lack of resources and support for detailed evaluation of effectiveness, and the challenges of capturing high quality and meaningful data in these settings, have been discussed elsewhere [27]. It was also difficult to determine whether the co-located service was the sole contributor to reported positive outcomes. Therefore, longitudinal studies using diverse methods to establish the contribution of community co-located services for improving mental health or preventing poor mental health over time, and the associated economic value of such approaches, are still required.

### Implications for policy makers and future research recommendations

Co-located services by community organizations stand out from statutory services due to their warmth, positive approach, and community trust. Importantly, these organisations provide an entry route to mental health support that is distinct from the formal health sector, thus widening the population we can reach even through established cross-sector partnerships such as social prescribing. Future commissioning and funding arrangements should not undermine these characteristics. It is important to acknowledge the critical role that the community sector plays in alleviating health inequalities through mechanisms of increasing access and empowerment and for this to be balanced against the need for measurement of performance. An over reliance on metric-based evaluations may lead to emphasis on maximising throughput and performance improvement, ultimately negating what it is that makes them work successfully. We need to consider how to capture reach and impact in a meaningful way beyond ‘tick box’ demographics and recommend working with delivery organisations and communities to co-produce these approaches.

Our findings show that although there is no one single model of community co-location, different approaches share the potential to positively impact public mental health. However, co-location does not always lead to integrated services [61], and it may be that community-to-community co-location works better than community-to-statutory co-location (because of a clash in underlying principles and ways of working). Irrespective of operational models, to maximise benefits it is important that there are good inter-service relationships, that the values of services and the populations that they seek to service are aligned and that there are structures in place that support co-working (e.g., regular cross service meetings). It may even be the case that good partnership working with service providers in other locations may be more effective for service delivery than working in the same building where there are poor inter-service relationships. This is in line with wider work that identified a shared purpose during the COVID-19 pandemic resulted in stronger relationships both within and across organisations in local delivery systems [62].

The biggest threats to the sustainability of these services may be from wider contexts. If funding streams can value, not constrain, these ways of working, this will provide more reliable access to funding (and enable services to have appropriate paid roles alongside voluntary elements). Importantly, this should not be via depleting mental health resources in clinical services or for crisis care – these are still vital and underfunded.

## Conclusion

Here we have shown how co-located services in community settings can positively impact public mental health and reduce mental health inequalities. In a sentence, providing holistic and person-centred care, reducing stigma, providing safe delivery environments, and overcoming barriers to accessibility widens the reach of services to those most in need, and encourages earlier and more consistent access to support.

### Recommendations

Policy makers and commissioners should develop and expand community-based co-located services, recognising the value of these community services, without constraining the ways in which they work. Theorists and researchers should examine the impact of community services on mental health, considering their economic value. Additionally, they should investigate optimizing service access by removing psychological barriers and enhancing trust. Expanding provision of community-based co-location and our understanding of its nuances will support more effective mental health promotion and prevention. This is especially important for groups facing multiple disadvantages or those who have been disengaged from traditional healthcare settings.

### Electronic supplementary material

Below is the link to the electronic supplementary material.

Supplementary Material 1

## Data Availability

Anonymised interviews transcripts are available from the authors on request. Please contact Fiona.duncan@newcastle.ac.uk.

## Abbreviations

UK: United Kingdom
RAMESES II: Realist and Meta-narrative Evidence Synthesis: Evolving Standards
COVID-19: SARS-COVID-19
CMO: Context-Mechanism-Outcome
IT: Information Technology
NHS: National Health Service
