# Supplementary material for: How co-locating public mental health interventions in community settings impacts mental health and health inequalities: a multi-site realist evaluation
Source: BMC Public Health. 2023 Dec 7;23:2445. doi: 10.1186/s12889-023-17404-x (PMC10702025; doi:10.1186/s12889-023-17404-x)
Supplement: Supplementary file 1 — Supplementary Material 1 [file 12889_2023_17404_MOESM1_ESM.docx]

**Additional file – Participant characteristics by case study site**

|  | **Role of participants (N)** | **Gender (N)** | **Age Group (years), (N)** | **Ethnicity * (N)** |
| --- | --- | --- | --- | --- |
| **Case Study Site 1** | Service providers (5) | Female (2)  Male (3) | Age data not available | Black or Black British African (4)  Arab (1) |
|  | Users of service (5) | Female (2)  Male (3) | 35-44, (3)  45-54, (2) | Black or Black British African (4)  British Asian (1) |
| **Case Study Site 2** | Service providers (5) | Female (4)  Male (1) | Age data not available | White British (4)  Other white (1) |
|  | Users of service (9) | Female (5)  Male (4) | 25-34, (1)  35-44, (1)  45-54, (3)  55-64, (4) | White British (1)  Black or Black British Caribbean (3)  Mixed white and black Caribbean (1)  British Asian (1)  Arab (1)  Other mixed (1)  Other (1) |
| **Case Study Site 3** | Service providers (5) | Female (3)  Male (2) | Age data not available | White British (5) |
|  | Users of service (5) | Female (2)  Male (3) | 25-34, (1)  35-44, (2)  55-64, (2) | White British (5) |
| **Case Study Site 4** | Service providers (6) | Female (4)  Male (2) | Age data not available | White British (6) |
|  | Users of service (8) | Female (3)  Male (5) | 18-24, (1)  45-54, (3)  55-64, (4) | British Asian (3)  White British (5) |
| **Case Study Site 5** | Service providers (6) | Female (4)  Male (2) | Age data not available | White British (6) |
|  | Members of site co-creation group (1) | Female (1)  Male (0) | Age data not available | British Asian (1) |
|  | Users of service (0) | N/A | N/A | N/A |
| **Case Study Site 6** | Service providers (5) | Female (3)  Male (2) | Age data not available | White British (5) |
|  | Users of service (5) | Female (2)  Male (3) | 25-34, (1)  45-54, (2)  55-64, (2) | Ethnicity data not available |

*Definition of ethnicity was based on categories from Census 2021 as defined by the United Kingdom’s Office for National Statistics
